# Idealized hydrodynamical numerical model dataset with no-river runoff at the western tropical North Atlantic

**DOI:** 10.12688/openreseurope.15747.1

**Published:** 2023-04-28

**Authors:** Humberto L. Varona, Julia Araujo, Moacyr Araujo, Marcus Silva

**Affiliations:** 1 Center for Risk Analysis, Reliability Engineering and Environmental Modeling, Federal University of Pernambuco, Recife, Pernambuco, 50740-550, Brazil; 2 Brazilian Research network on Global Climate Change (Rede CLIMA), INPE, São José dos Campos, São Paulo, 12227-010, Brazil

**Keywords:** Amazon River, Western tropical Atlantic, No-River runoff, Hydro-thermodynamics, Dataset, ROMS model, NetCDF, Matlab

## Abstract

The western tropical North Atlantic (WTNA) is a very complex region, with the influence of intense western boundary currents in connection with equatorial zonal currents, important atmospheric forcings (e.g Intertropical Convergence Zone), mesoscale activities (e.g NBC rings), and the world’s largest river discharge as the Amazon River runoff. The volume discharge is equivalent to more than one-third of the Atlantic river freshwater input, with a plume that spreads over the region reaching the northwestward Caribbean Sea and eastward longitudes of 30°W, and influencing from physical to biological structures. Therefore, in order to enable and encourage more understanding of the region, here we present a dataset based on an idealized scenario of no river runoff of the Amazon River and Par ´a River in the WTNA. The numerical simulations were conducted with a regional oceanic modeling system (ROMS) model and three pairs of files were generated with the model outputs: (i) ROMS-files, with the parameters of the ROMS-outputs raw data in a NetCDF format and monthly and weekly frequencies; (ii) MATLAB-files, which contain oceanographic parameters also in monthly and weekly frequencies; and (iii) NetCDF-files, with oceanographic parameters again in monthly and weekly frequencies. For each file, we present the coordinates and variable names, descriptions, and correspondent units. The dataset is available in the Science Data Bank repository (doi: https://doi.org/10.57760/sciencedb.02145)

## Plain language summary

The dataset here presented is very useful for oceanographers, marine biologists, and chemists in an idealized scenario with no river runoff (Amazon River and Pará River) in the western tropical North Atlantic (WTNA). You can study the ocean properties (for example, temperature, salinity, and currents), the influence of the chemical components (for example, nutrients and CO2 fluxes), and the biological relations of different organisms and their distribution at the region. With these simulation results, you can compare the observed data you are working on with our numerical ocean model outputs, estimating the influence of the Amazon and Pará Rivers in this region. These data are stored in the following formats: ROMS model NetCDF, Matlab, and NetCDF standard. In the case of Matlab and NetCDF standard, no pre-processing is required. Moreover, the dataset is available in the open repository of Science Data Bank (doi: 10.57760/sciencedb.02145), and to access click here.

## Introduction

The western tropical North Atlantic (WTNA) is characterized as an extremely complex region with the influence of different physical forcings, such as strong western boundary currents ( *e.g*. North Brazil Undercurrent and North Brazil Current) in connection with zonal equatorial currents ( *e.g* South Equatorial Undercurrent, Equatorial Undercurrent, and North Equatorial Countercurrent) as part of the equatorial current system ^1^; important atmospheric drivers ( *e.g* trade winds and the Intertropical Convergence Zone) ^2^; and mesoscale activity ( *e.g* NBC rings) ^3, 4^. Besides, the region also presents an important pathway of interhemispheric transport of mass, heat, and salt as part of the Atlantic Meridional Overturning Circulation ^5, 6^.

A singular aspect of this region is the presence of the Amazon River runoff, the world’s largest river discharge accounting for 18% of the continental water flow to the oceans and approximately 32% of the freshwater input to the Atlantic ocean, with an average of 222,800 m ^3^ s ^−1^ ^7, 8^. The discharge volume reaches up to two-fold the net atmospheric freshwater flux (evaporation-precipitation) over the WTNA ^9^. Furthermore, the Amazon River forms a plume over the region, which extends over a thousand of kilometers, flowing northwestward and reaching the Caribbean Sea and also retroflecting eastward through the North Equatorial Countercurrent and reaching latitudes of 30°W ^10, 11^. Therefore, the region is marked by an intense land-ocean synergy, with several processes in play, such as mixed layer depth changes and formation of barrier layers, leading variations of the ocean surface heat balance ^12, 13^; high terrestrial sediments and dissolved organic matter transport ^11^; and complex and important biogeochemical interactions, from nutrient and carbon fluxes ^14, 15^ to changes on biological community structures ^16^.

The present work brings a dataset originated from simulations using the regional oceanic modeling system (ROMS) model in an idealized scenario of no river runoff of the Amazon River and the Pará River in the WTNA. The main goal for the creation of the here presented dataset is to enable and encourage the development of more researches in the region to advance in bridging the knowledge gaps of a such complex area. In the following Sections, we present the methods, which introduces the study area and explains the model configuration, the initial and boundary parameters; the dataset, with the directory structure of the dataset, the generated files, and details of the coordinates and variables of each of the generated files; and lastly, the data availability.

## Methods

**Figure 1.**
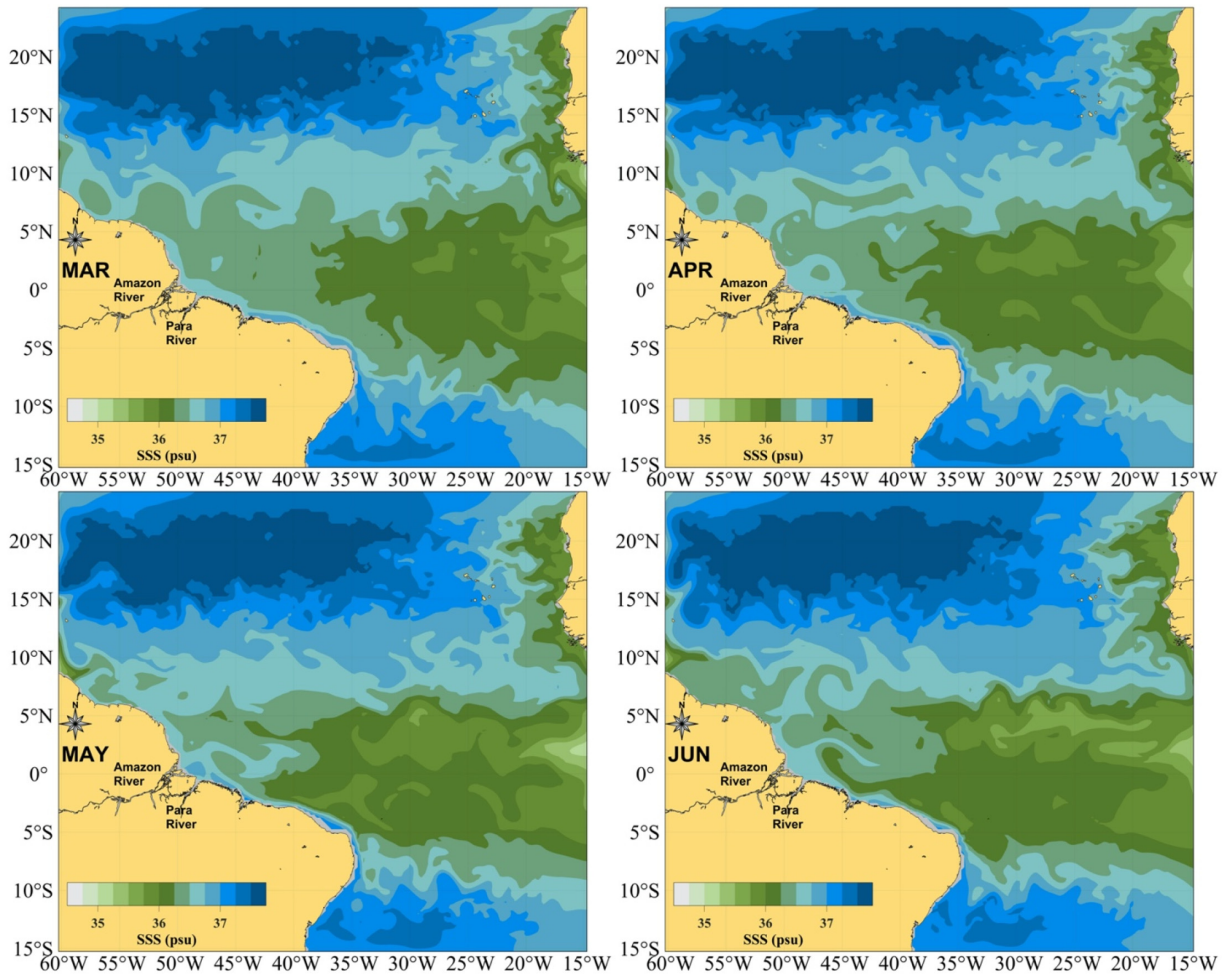
Geographic location of the dataset. Sea Surface Salinity field in the periods of maximum Pará River runoff (March–April, upper panels) and maximum Amazon River runoff (May–June, lower panels).

This study was conducted through ideal simulations with the ROMS numerical model, using the ROMS-AGRIF version (regional ocean modeling system - adaptive grid refinement) ^17– 20^, which has been successfully tested in the WTNA, specifically off the North and Northeastern Brazilian coast ^12, 21– 31^. The geographic localization of this dataset is 60.5°-24°W/5°S-16°N, which is illustrated in Figure 1 with the monthly sea surface temperature (SST) horizontal distribution in the period maximum Pará River runoff (March-April) and maximum Amazon River runoff (May-June) ^7^. In these four months, the minimum SST found in the area corresponding to this dataset is 35 psu (practical salinity unit), with values reaching 32 psu over the river plume and 27 psu off the Amazon River mouth ^29^.

For our simulation, all initial and boundary parameters have been spatially discretized at a horizontal resolution of 0.25° with non-isotropic grid from Earth Topography v2 (ETOPO2) bathymetry ^32^ and vertically using the sigma coordinate system (32 vertical levels). In the configuration file (ROMSTOOLS; romstools_param.m), ROMSTOOLS is a set of Octave compatible Matlab functions used to perform pre-processing of ROMS model input data, we used theta_s = 6 and theta_b = 0 (These two parameters indicate to the ROMS model how it has to consider the distribution of vertical levels near the surface and the bottom, both must take values between zero and seven, the higher the values of these, the more vertical levels will be concentrated near the surface and the bottom) ^19, 33^, obtaining 12 and 20 levels in the first 100m and 500m depth, respectively. Other parameters used in the grid discretization are: hc = 5.0, hmin = 150, hmax_coast = 500, hmax = 5000, rtarget = 0.25 (slope), and Roa = 85e3.

Since tidal influence is important for the seawater mixing process in the surface layers, tides have been taken into account in our simulation and data were obtained from OSU TPXO7 Tide model ^34, 35^. The North, South, East and West lateral boundaries were considered open and the sea surface salinity (SSS) and sea surface temperature (SST) values for initial and boundary conditions were restricted to monthly mean values obtained from the World Ocean Atlas 2009 (WOA2009 dataset) ^36, 37^. For the surface forcing, it was used monthly climatology of the Comprehensive Ocean-Atmosphere Data Set (COADS05 dataset) ^38^. This dataset is the last year of the numerical output (roms_his.nc and roms_avg.nc) obtained by a ROMS-model simulation of 11 years, and it was called the No-River Runoff experiment (NRF) in 27, 29.

## Dataset

**Figure 2.**
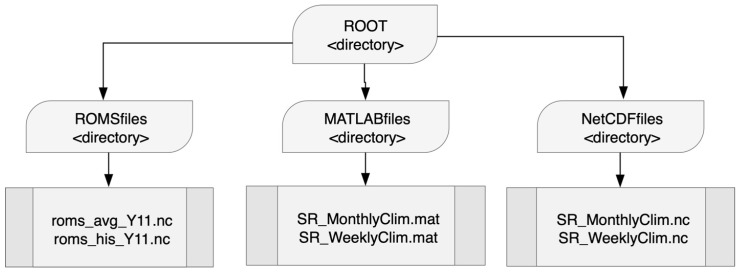
Distribution of files by directory according to their format.

The idealized hydrodynamical numerical model database with no-river runoff in the Northern and Northeastern Brazilian coast (IHMD-NRF) is the result of a research carried out by 29. As shown in Table 1, the dataset includes three pairs of files: (i) ROMS-files as two files in ROMS NetCDF format, containing the monthly and weekly numerical output (raw data); (ii) MATLAB-files as two files processed and converted in Matlab; and (iii) NetCDF-files as two more files also processed and converted in NetCDF standard format with mNC tool ^39^, the file pairs in (ii) and (iii) are identical to (i), but have been converted to Matlab formats (they can be read in Octave, R and Python) and the NetCDF standard. For each file, some metadata were added using freeware tools called Climate Data Operator (CDO) ^40^ and NetCDF Operators (NCO) ^41^. All files are organized in the directory structure shown in Figure 2.

**Table 1.** Files that integrate this dataset and its main characteristics.

Filename	Description	Format	Processing level	Size
roms_his_Y11.nc	**ROMSfile** of monthly climatology of the ROMS simulation	ROMS NetCDF	Raw data	351 Mb
roms_avg_Y11.nc	**ROMSfile** of weekly climatology of the ROMS simulation	ROMS NetCDF	Raw data	1.4 Gb
SR_MonthlyClim.mat	**MATLABfile** of monthly climatology of the ROMS simulation	Matlab	Processed data	694 Mb
SR_WeeklyClim.mat	**MATLABfile** of weekly climatology of the ROMS simulation	Matlab	Processed data	2.7 Gb
SR_MonthlyClim.nc	**NetCDFfile** of monthly climatology of the ROMS simulation	NetCDF standard	Processed data	1.1 Gb
SR_WeeklyClim.nc	**NetCDFfile** of weekly climatology of the ROMS simulation	NetCDF standard	Processed data	4.5 Gb

The *ROMSfiles* directory stores the ROMS model numerical output files for monthly and weekly data for the 11 ^th^ year.

The outputs’ dimensions, their description and dimension size are listed in Table 2. The parameter *time* does not need any conversion when working with MATLAB, the function *datestr* provides the date. The monthly output is dated on the first day of each month, while the weekly output is on the 4 ^th^, 12 ^th^, 19 ^th^ and 27 ^th^ of each month. The year of this date is relative to the simulation year, so it will always be 0011 (11 ^th^ year). Table 2 shows the *time* parameter twice, once at monthly and once at weekly frequency, corresponding to each of the ROMS-files outputs. In the monthly output file ( *rooms_his_Y11.nc*), there are 13 values because January - year 0012 is also included.

**Table 2.** ROMS-files dimensions (coordinate system parameters).

Dimensions	Description	Variable dimension
xi_rho	Longitude dimension for scalar variables	185
eta_rho	Latitude dimension for scalar variables	161
s_rho	Vertical levels for all variables	32
time	Time values, weekly climatology	48
time	Time values, monthly climatology	13
xi_u	Longitude dimension for vector variable	184
eta_v	Latitude dimension for vector variable	160

The current velocity component (vector) grids have 184 × 160 nodes, while the scalar parameter grids have 185 × 161 nodes. Still for the ROMSfiles, Table 3 shows all the variables, which are related to the oceanographic and surface physical parameters. Other complementary descriptions of the ROMS model output files, *i.e* ROMS-files, can be found in 42, 43. All the ROMS model output files that are in the directory can be visualized with the tools that are in the directory called *Roms_tools/Visualization_tools/*. The folder is a component of ROMSTOOLS, the main script is roms_gui and it is a graphical user interface (GUI) ^19, 33^. This GUI is developed in MATLAB GUIDE (Octave-compatible), so it allows you to reuse other functions to retrieve parameters at a given level or depth, or even at a specific coordinate or date, *e.g*.: get_var.m.

**Table 3.** ROMSfiles variables (oceanographic and surface parameters) contained in the *ROMSfiles* directory.

Variables	Description	Unit
lon_rho	Horizontal nodes longitude	Degrees east
lat_rho	lat_rho Horizontal nodes latitude	Degrees north
lon_u	Horizontal nodes longitude	Degrees east
lat_u	Horizontal nodes latitude	Degrees north
lon_v	Horizontal nodes longitude	Degrees east
lat_v	Horizontal nodes latitude	Degrees north
h	Bathymetry	m
temp	Potential temperature	°C
salt	Salinity	PSU
u	u-momentum component	m s ^−1^
v	v-momentum component	m s ^−1^
w	Vertical momentum component	m s ^−1^
zeta	Sea Surface Height	m
ubar	Vertically integrated u-momentum component	m s ^−1^
vbar	Vertically integrated v-momentum component	m s ^−1^
f	Coriolis parameter	s ^−1^
bostr	Kinematic bottom stress	N s ^−1^
wstr	Kinematic wind stress	N s ^−1^
sustr	Kinematic u wind stress component	N s ^−1^
svstr	Kinematic v wind stress component	N s ^−1^
diff3d	Horizontal diffusivity coefficient	-
hbl	Depth of planetary boundary layer	m
hbbl	Depth of bottom boundary layer	m
shflux	Surface net heat flux	W m ^−2^
swflux	Surface freshwater flux (E-P)	cm day ^−1^
swrad	Short-wave surface radiation	W m ^−2^
swrad	Short-wave surface radiation	W m ^−2^
mask_rho	Land mask (0 to land, 1 to ocean)	-
mask_u	Land mask (0 to land, 1 to ocean)	-
mask_v	Land mask (0 to land, 1 to ocean)	-

The two pairs of files, *i.e* MATLAB-files and NetCDF-files, contain oceanographic parameters such as salinity, temperature, sea currents, sea surface height (SSH), seawater density, and vorticity ( Table 4 and Table 5). Here we present the descriptions of the oceanographic parameters in standard MATLAB and NetCDF formats (stored in the *MATLABfiles* and *NetCDFfiles* directories, respectively), all these files can be read in ncview, Panoply, CDO, NCO and other freewares like Octave, R and Python. In both cases, the *time* parameter has dimension size of 12 for each month of the 11 ^th^ year of the monthly simulation and it also presents dimension size of 48 for the weekly simulation, with output on the same dates as the equivalent files in NetCDF ROMS format (ROMS-files). In these four pairs of files, the vector and scalar data grids are referenced with the same coordinate system.

**Table 4.** MATLAB-files variables (oceanographic parameters) contained in the *MATLABfiles* directory.

Variables	Description	Unit
Lons	Horizontal nodes longitude	Degrees east
Lats	Horizontal nodes latitude	Degrees north
Time	Time	Months
DEPTHS	Depths	m
bathymetry	Bathymetry	m
temp	Potential temperature	°C
salt	Salinity	PSU
ssh	Sea Surface Height	m
u	u-momentum component	m s ^−1^
v	v-momentum component	m s ^−1^
rho	Seawater density	Kg m−3
currspd	Current speed	m s ^−1^
vort	Vorticity	s ^−1^

**Table 5.** NetCDF-files variables (oceanographic parameters) contained in the *NetCDFfiles* directory.

Variables	Description	Unit
lon	Horizontal nodes longitude	Degrees east
lat	Horizontal nodes latitude	Degrees north
time	Time	Months
depth	Depths	m
h	Bathymetry	m
temp	Potential temperature	°C
salt	Salinity	PSU
ssh	Sea Surface Height	m
ucurr	u-momentum component	m s ^−1^
vcurr	v-momentum component	m s ^−1^
swd	Seawater density	Kg m ^−3^
cspd	Current speed	m s ^−1^
vort	Vorticity	s ^−1^

The standard NetCDF files, *i.e* NetCDF-files, are self-describing as it is possible to explore a very detailed description of the metadata with the *ncdump* command in terminal ^44^. You can also preview the data using the *ncview* command in terminal ^45^, GrADS ^46^ and Panoply data viewer ^47^, the latter depending on the Java Runtime Environment.

## Data Availability

Science Data Bank: Idealized hydrodynamical numerical model database with no river runoff in the northeastern and northern regions of Brazil (IHMD-NRF) https://doi.org/10.57760/sciencedb.02145 This project contains the following underlying data: - roms_his_Y11.nc (Monthly climatology raw data) - roms_avg_Y11.nc (Weekly climatology raw data) Science Data Bank: Idealized hydrodynamical numerical model database with no river runoff in the northeastern and northern regions of Brazil (IHMD-NRF) https://doi.org/10.57760/sciencedb.02145 This project contains the following underlying data: - SR_MonthlyClim.mat (Monthly climatology of ROMS simulation in MATLAB format) - SR_WeeklyClim.mat (Weekly climatology of ROMS simulation in MATLAB format) - SR_MonthlyClim.nc (Monthly climatology of ROMS simulation in NetCD format) - SR_WeeklyClim.nc (Weekly climatology of ROMS simulation in NetCD format) Data are available under the terms of the Creative Commons Attribution 4.0 International license (CC-BY 4.0).
