# Supplementary material for: Idealized hydrodynamical numerical model dataset with no-river runoff at the western tropical North Atlantic
Source: Open Res Eur. 2023 Apr 28;3:67. [Version 1] doi: 10.12688/openreseurope.15747.1 (PMC10446047; doi:10.12688/openreseurope.15747.1)
Supplement: Peer review history

# Peer review: Reviewer response for version 1

**DOI:** 10.21956/openreseurope.17011.r31415

**Published:** 2023-06-01

**Authors:** Sandrine Djakouré

**Affiliations:** 1 Laboratory of Matter Sciences, Environment and Solar Energy (LASMES), University Felix Houphouet Boigny of Cocody-Abidjan, Abidjan, Cote d'Ivoire

This data note presents a dataset *“originated from simulations using the regional oceanic modeling system (ROMS) model in an idealized scenario of no river runoff”* **. **The study mainly conducted from modeling products aims to provide a database of oceanographic parameters useful for studies of the dynamics of the complex western tropical North Atlantic (WTNA) Ocean. The methods and materials provided all the information needed to reproduce this simulation. This dataset could be used for comparative studies to analyze the influence of the Amazon River runoff, the world’s largest river discharge particularly in biogeochemistry and on ocean-atmosphere interactions. This database is a welcome addition. The report is well written, the figures are clear. Nevertheless, I suggest a few changes that should improve this data note.

Minor Comments:

**Methods section:**

- In the text *“This dataset is the last year of the numerical output (roms_his.nc and roms_avg.nc) obtained by a ROMS-model simulation of 11 years, and it was called the No-River Runoff experiment (NRF) in 27, 29”.*
  What about the spin-up? Why did you choose the ROMS-model simulation of 11 years? Can you give more arguments to justify your choice?
- CROCO is a free surface model; therefore the model conserves the total mass of salt in the ocean so as to control the salinity in the model. It is an idealized scenario of no-river runoff but I suggest inserting few words about the salinity “recall”.

Specific comments

**Methods section: **

- Lines 7 “which is illustrated in Figure 1 with the monthly sea surface temperature (SST) horizontal”. There is a mistake. It is not sea surface temperature but sea surface salinity.

**References**

- ***“**21. Silva AC, Bourles B, Araujo M: Circulation of the thermocline salinity maximum waters off the northern brazil as inferred from in situ measurements and numerical results. Ann Geophys. 2009; 27(5): 1861–1873.”*
  Please add the circumflex accent “Bourlès”.

Are sufficient details of methods and materials provided to allow replication by others?

Yes

Is the rationale for creating the dataset(s) clearly described?

Yes

Are the datasets clearly presented in a useable and accessible format?

Yes

Are the protocols appropriate and is the work technically sound?

Yes

Reviewer Expertise:

Physical Oceanography, Numerical modeling (CROCO), ocean-atmosphere interactions, biogeochemistry.

I confirm that I have read this submission and believe that I have an appropriate level of expertise to confirm that it is of an acceptable scientific standard.

# Peer review: Reviewer response for version 1

**DOI:** 10.21956/openreseurope.17011.r31414

**Published:** 2023-05-16

**Authors:** Marcio M. Cintra

**Affiliations:** 1 Departamento de Ciências Atmosféricas e Climáticas, DCAC, Universidade Federal do Rio Grande do Norte, Natal, State of Rio Grande do Norte, Brazil

The article has as main goal to provide a database of ocean variables obtained from a regional numerical simulation developed for the western tropical Atlantic Ocean without considering the influence of river discharge from the Amazon River. This approach is interesting because it facilitates the work for studies that come, complement, refine, and better detail processes in this region, especially those dedicated to measuring the influence of the river on a given subject.

 The article is very concise and well written. The methodology employed is totally adequate to what is proposed and allows the reproduction of the experiment. I only suggest that it be inserted in the methodology which tidal components were used in the simulation. Furthermore, everything is well placed and there are no questions.

 I tested the files to verify if everything that was described is working properly. And yes, everything is perfect. Therefore, my recommendation is that it be indexed.

Are sufficient details of methods and materials provided to allow replication by others?

Yes

Is the rationale for creating the dataset(s) clearly described?

Yes

Are the datasets clearly presented in a useable and accessible format?

Yes

Are the protocols appropriate and is the work technically sound?

Yes

Reviewer Expertise:

Physical Oceanography, Numerical modeling, ocean-atmosphere interactions,

I confirm that I have read this submission and believe that I have an appropriate level of expertise to confirm that it is of an acceptable scientific standard.
